# An Unusual Cause of Chest Pain in a Pediatric Patient on Hemodialysis

**DOI:** 10.34067/KID.0000000676

**Published:** 2025-05-29

**Authors:** Eva Glenn Lecea, Kristen Favel

**Affiliations:** Division of Nephrology, Department of Pediatrics, University of California in San Francisco, San Francisco, California

**Keywords:** chronic hemodialysis, hemodialysis access, hemodialysis hazards

## Abstract

**Figure:**
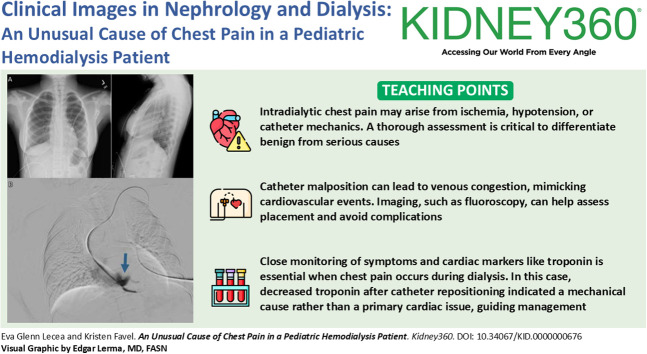

## Case Presentation

**Figure 1:**
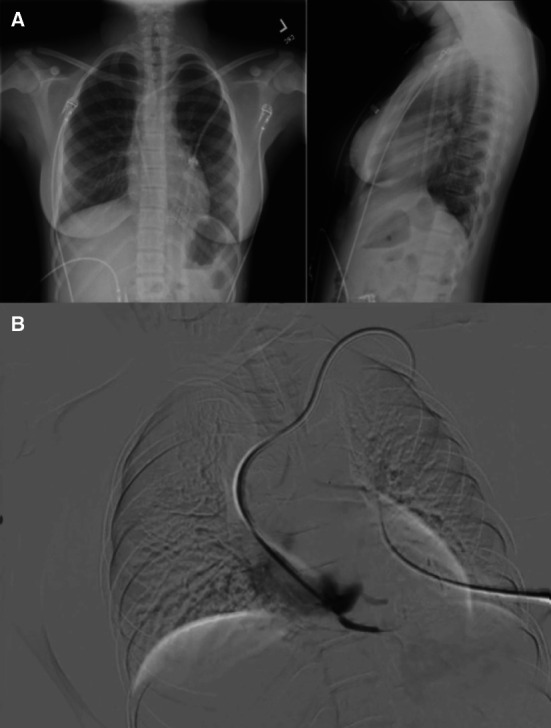
**Chest x-rays and radiology-guided fluoroscopic study of hemodialysis catheter.** (A) Anteroposterior and lateral chest x-rays showing the position of the tip of the left internal jugular vein catheter. (B) Interventional radiology–guided fluoroscopic study of the hemodialysis catheter showing contrast refluxing into the coronary vein (arrow).

A 14-year-old adolescent girl with ESKD from a cloacal anomaly and obstructive uropathy presented with chest pain during hemodialysis. Her history included multiple surgeries, notably a double aortic arch repair, and she began hemodialysis at age 9 years. Her medications were amlodipine, sodium zirconium cyclosilicate, sucroferric oxyhydroxide, and paricalcitol (Figure 1).

During hemodialysis, she experienced chest pain, nausea, vomiting, and hypotension. Her pretreatment weight was 41.5 kg (dry weight 39.8 kg) and BP 110/64 mm Hg; and after 2 hours of hemodialysis, she developed sharp midsternal chest pain (8/10), lightheadedness, nausea, and emesis. Her BP dropped to 77/44 mm Hg, and heart rate was 65 bpm. Partial relief was achieved with acetaminophen, ondansetron, and intravenous saline, but dialysis was stopped when symptoms recurred, leading to an emergency department referral.

In the emergency department, she had an elevated troponin of 0.63 ng/ml, with no other significant laboratory abnormalities except for her baseline elevated creatinine concentration and mild anemia. Chest x-ray and electrocardiogram were normal. Pediatric cardiology did not pursue further intervention given a normal echocardiogram 5 months earlier. On reattempting hemodialysis the next day, chest pain returned.

Fluoroscopy by interventional radiology revealed that her left internal jugular hemodialysis catheter terminated in the right atrium, causing venous reflux into a coronary vein (Figure 1). The catheter was repositioned in the right atrium, leading to a gradual reduction in chest pain and normalization of troponin levels. She had no further issues with hemodialysis before discharge.

## Discussion

Chest pain during dialysis is complex, potentially signaling acute coronary syndrome, aortic dissection, or pulmonary embolism.^1^ Pediatric patients on hemodialysis, while generally free from typical coronary artery disease, face cardiovascular risks, such as endothelial dysfunction and early atherosclerosis, due to uremia. Intradialytic hypotension, affecting 20%–30% of sessions, can lead to myocardial stunning and possibly heart failure if recurrent.^2,3^ Inadequate cardiac output, ischemia, or diastolic dysfunction may also manifest as chest pain or hypotension.^1^

In this case, chest pain was likely due to hemodialysis catheter placement, which increased coronary vein pressure, impairing myocardial perfusion. This mechanism resembles coronary sinus occlusion, where elevated transcapillary pressure can cause myocardial injury.^4^

## Teaching Points

- Intradialytic chest pain may arise from ischemia, hypotension, or catheter mechanics. A thorough assessment is critical to differentiate benign from serious causes.
- Catheter malposition can lead to venous congestion, mimicking cardiovascular events. Imaging, such as fluoroscopy, can help assess placement and avoid complications.
- Close monitoring of symptoms and cardiac markers, such as troponin, is essential when chest pain occurs during dialysis. In this case, decreased troponin after catheter repositioning indicated a mechanical cause rather than a primary cardiac issue, guiding management.
